# Draft Genome Sequences of *Sphingomonas* Species Associated with the International Space Station

**DOI:** 10.1128/MRA.00578-20

**Published:** 2020-06-18

**Authors:** Swati Bijlani, Nitin K. Singh, Christopher E. Mason, Clay C. C. Wang, Kasthuri Venkateswaran

**Affiliations:** a University of Southern California, Los Angeles, California, USA; b Jet Propulsion Laboratory, California Institute of Technology, Pasadena, California, USA; c The WorldQuant Initiative for Quantitative Prediction, Weill Cornell Medicine, New York, New York, USA; University of Maryland School of Medicine

## Abstract

The draft genome sequences of three *Sphingomonas* strains isolated from the International Space Station (ISS) were assembled. These genomic sequences will help in understanding the influence of microgravity conditions on their potential bioactive compound production and other important characteristics compared to their Earth counterparts.

## ANNOUNCEMENT

*Sphingomonas* species have been isolated from a variety of habitats, and some species possess the unique capability to degrade pollutants (1). Some of the species are known for biofilm formation and eventually corrode the metal surfaces (2), production of bioactive compounds (3), and plant-pathogenic characteristics (4). Sphingomonas paucimobilis has been reported to be associated with infections in immunocompromised patients (5, 6).

In an ongoing microbial observatory experiment, several microbial strains were isolated from the International Space Station (ISS) (7). The generation of whole-genome sequences (WGS) to enable the comparative genomic characterization of ISS *Sphingomonas* species with their Earth counterparts would lead to the identification of the genetic determinants potentially responsible for their important characteristics due to microgravity and elevated radiation conditions.

The WGS belonging to two Sphingomonas sanguinis strains and one *S. paucimobilis* isolate were assembled into scaffolds. Sample collection, processing, and presumptive identification of these isolates based on 16S rRNA gene sequences were published elsewhere (7). Briefly, samples collected from the ISS were processed, and 100 μl of each dilution was plated on Reasoner’s 2A (R2A) agar. The plates were incubated at 25°C for 7 days. The single colony obtained was restreaked onto R2A plates and incubated at 25°C for 3 days. A biomass of approximately 1 μg wet weight was collected for each strain and pooled for DNA extraction. Total nucleic acid extraction was carried out using a ZymoBIOMICS 96 MagBead DNA kit (lysis tubes; Zymo Research, USA) after bead beating using a Bertin Precellys instrument. This was followed by library preparation using the Nextera Flex protocol as per Illumina document number 1000000025416 v07. The initial amount of DNA for library preparation was quantified, and depending on the input DNA concentration, 5 to 12 cycles of PCR were carried out to normalize the output. The amplified genomic DNA fragments were indexed and pooled in 384-plex configuration. Whole-genome shotgun sequencing was performed on a NovaSeq 6000 S4 flow cell in paired-end (PE) 2 × 150-bp format. The data were filtered with NGS QC Toolkit v2.3 (8) for high-quality (HQ) vector- and adaptor-free reads for genome assembly (cutoff read length for HQ, 80%; cutoff quality score, 20). The number of filtered reads obtained (Table 1) was used for assembly with the SPAdes v3.14.0 (9) genome assembler (k-mer size, 32 to 72 bases). The genome was annotated using the NCBI Prokaryotic Genome Annotation Pipeline (PGAP) v4.11 (10, 11). Default parameters were used for all software except where otherwise noted.

**TABLE 1:** Summary of the draft whole-genome sequences of three *Sphingomonas* strains isolated from the ISS

Species and strain	NCBI accession no.	Isolation location	No. of scaffolds	Genome size (bp)	*N*_50_ (bp)	Median coverage (×)	G+C content (%)	No. of raw reads (millions)	No. of filtered reads used for assembly (millions)	No. of coding sequences
*Sphingomonas sanguinis* IIF7SW-B3A	JABEOW000000000.1	Lab 3 overhead	53	3,989,786	149,509	1,102	66.23	30.91	30.67	3,659
*Sphingomonas sanguinis* IIF7SW-B5	JABEOV000000000.1	Lab 3 overhead	51	4,398,996	375,188	916	66.08	28.3	28.1	4,047
*Sphingomonas paucimobilis* FKI-L5-BR-P1	JABEOU000000000.1	KSC-PHSF^*a*^ cleanroom floor	73	4,572,738	138,969	448	65.53	14.27	14.18	4,182

a KSC-PHSF, Kennedy Space Center Payload Hazardous Servicing Facility.

The details of the final assembly are shown in Table 1. The phylogenetic affiliations of the strains isolated in this study were confirmed based on the similarity of the 16S rRNA gene sequences extracted from the genomes (12) and the average nucleotide identity (13). The average nucleotide identities (ANIs) of the queried genomes were calculated using EzBioCloud (14) with their corresponding type strains.

### Data availability.

The WGS and raw data are deposited under BioProject accession number PRJNA629834. The WGS accession numbers are listed in Table 1. The WGS was also deposited at GeneLab (GeneLab data set GLDS-298, https://genelab-data.ndc.nasa.gov/genelab/accession/GLDS-298/). The version described in this paper is the first version.
